# Enhanced nematicidal potential of the chitinase *pachi* from *Pseudomonas aeruginosa* in association with *Cry21Aa*

**DOI:** 10.1038/srep14395

**Published:** 2015-09-24

**Authors:** Lin Chen, Huang Jiang, Qipeng Cheng, Junpeng Chen, Gaobing Wu, Ashok Kumar, Ming Sun, Ziduo Liu

**Affiliations:** 1 State Key Laboratory of Agricultural Microbiology, College of Life Science and Technology, Huazhong Agricultural University, Wuhan 430 070, China; 2 State Key Laboratory of Agricultural Microbiology, College of Plant Science and Technology, Huazhong Agricultural University, Wuhan 430070, China

## Abstract

Nematodes are known to be harmful to various crops, vegetables, plants and insects. The present study reports that, chitin upregulates the activity of chitinase (20%) and nematicidal potential (15%) of *Pseudomonas aeruginosa*. The chitinase gene (*pachi*) from *P. aeruginosa* was cloned, and its nematicidal activity of pachi protein against *Caenorhabditis elegans* was studied. The mortality rate induced by pachi increased by 6.3-fold when in association with Cry21Aa from *Bacillus thuringiensis*. Pachi efficiently killed *C. elegans* in its native state (LC_50_ = 387.3 ± 31.7 μg/ml), as well as in association with Cry21Aa (LC_50_ = 30.9 ± 4.1 μg/ml), by degrading the cuticle, egg shell and intestine in a relatively short time period of 24 h. To explore the nematidal potential of chitinase, six fusion proteins were constructed using gene engineering techniques. The CHACry showed higher activity against *C. elegans* than others owing to its high solubility. Notably, the CHACry showed a synergistic factor of 4.1 versus 3.5 a mixture [1:1] of pachi and Cry21Aa. The present study has identified eco-friendly biological routes (*e.g.,* mixed proteins, fusion proteins) with potent nematicidal activity, which not only can help to prevent major crop losses but also strengthen the agro-economy and increase gross crop yield.

Nematodes infect the root, stem and leaves of cultural crops, leading to plant wilting, chlorosis, reduced growth and ultimately death, resulting in major economic losses. In order to control nematodes, various chemicals have been used worldwide. These chemicals not only are toxic to the plants but also have various harmful effects on human health and the environment^1^. Gene engineering has opened new gateways to develop nontoxic bio-control agents that are effective in protecting from nematodiasis^2^. In the study of bio-control of nematodiasis, *C. elegans* has been used widely as an important model organism owing to its genetic manipulability and short life cycle^3^,^4^. A literature survey revealed that some nematophagous bacteria were effective in killing nematodes. For example, *Bacillus thuringiensis*, *Pseudomonas* and *Xenorhabdus nematohilus*, found in the rhizosphere of plants, showed high nematicidal activity^5^,^6^. The major virulence factors of nematophagous bacteria included microbial toxins, insecticidal crystal proteins (ICPs), hydrolytic enzymes and secondary metabolites, which killed the nematode(s) by various mechanisms. These proteins/enzymes acted on different body parts (cuticle, egg shell and intestine) and organs^7^,^8^. ICP was an important bio-control factor of *Bacillus thuringiensis* which has been used widely in bio-control of nematodiasis. The ICP was dissolved in the intestine and its C-terminal domain was cleaved by proteases to release the active region of the toxin^9^. The activated toxin was located at the brush border membrane vesicles and bound to its receptors, leading to epidermal cell rupture and pathogenic organism death^10^. In this study, Cry21Aa was tested on *C. elegans* and it showed higher nematicidal activity than Cry6Aa, Cry55Aa and Cry5Ba which have been reported as nematicidal factors^11^. The Cry21Aa consisted of N-terminal extension region, region of endotoxin N (14.9 kDa), region of delta-endotoxin C (28.8 kDa), and the C-terminus extension region. The toxin region of Cry21Aa (Cry) was composed of N-terminal extension region, endotoxin N and delta-endotoxin C. Chitinase was another important toxicity factor in bio-control of nematodiasis. Nematicidal chitinases were reported to be produced by various fungi, including *Monacrosporium thaumasium*^12^ and *Clonostachys rosea*^13^, which killed nematodes via egg shell digestion and cuticle hydrolysis^7^. As chitin was the main component of the egg shell and cuticle of nematodes and acted as a target for these nematicidal factors^14^, chitin and chitosan were incorporated into the soil to reduce nematode infection by inducing rhizobacteria to produce chitinase^15^. *Pseudomonas* was an important rhizobacteria strain, which could produce some hydrolases (protease and chitinase) in killing nematodes^16^,^17^,^18^.

To improve the nematicidal activity, many studies have been conducted on the synergistic effect(s) of two or more toxins against nematodes. For example, the cumulative effect of the Cry6A and Cry5B proteins [4:1] against *C. elegans* was significantly higher than their individual effects^19^. In another study, Cry6Aa, Cry55Aa and Cry5Ba were used in different sets and the Cry6Aa-Cry55Aa showed highest activity against *M. incognita*^11^. Recently, Luo *et al.* used the protease bmp1 as a synergistic factor to increase Cry5B activity (of ~7.9-fold) against *C. elegans*^20^. Moreover, not only mixed proteins but also fusion proteins have been widely applied as bio-control agents in agri-biotechnology to control pests. To improve the toxicity against adult aphids (*M. persicae*), the chitinase Bbchit1 was fused with the protease Pr1A, and the result showed that insecticidal activity has been increased 2 times compared to Bbchit1^21^. Additionally, the chitinase gene from tobacco (*Nicotiana tabacum*) was fused with *cry1Ac* gene from *Bacillus thuringiensis* and the insecticidal activity showed a remarkable increase of 11.3 times^22^.

While chitinase has been used widely in increasing ICP insecticidal activity, there are few reports about the use of chitinase as a factor in increasing ICP nematicidal activity. The objectives of the present study were (**i**) to investigate the synergistic effect of chitinase and ICP and (**ii**) to enhance the nematicidal activity by mixing or fusing the pachi from *P. aeruginosa* with Cry21Aa (Cry) to test their effects on eggs, larvae and mature nematodes and to explore the mode of action of nematicidal potential as a bio-control agent.

## Results

### Cloning chitinase pachi

**Figure 1:**
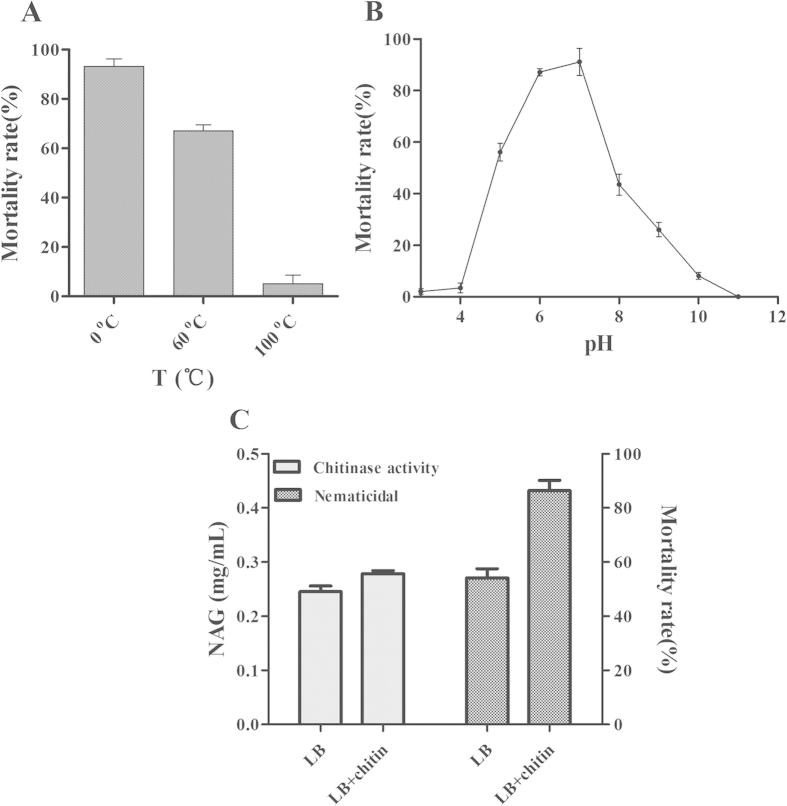
Chitinase as the potential toxin of *P. aeruginosa* against *C. elegans*. (**A**) Effects of different temperatures on nematicidal activity *P. aeruginosa*. (**B**) Effects of different acidic and alkaline conditions on nematicidal activity of *P. aeruginosa*. (**C**) Effects of chitin on the chitinase activity and nematicidal activity of *P. aeruginosa*. The concentration of *N-*acetylglucosamine (NAG) indicated chitinase activity. Nematicidal activity was measured using L4 worms (~80 worms each well) at 20 °C.

In this study, *P. aeruginosa* was selected for the bio-control of nematodiasis, using *C. elegans* as the model organism. The supernatant of lysate cell lost its nematicidal activity at high temperature, and under high acidic or alkaline conditions (Fig. 1A,B), which indicated that proteins were related to the nematicidal activity of *P. aeruginosa*. Previous work has shown that *P. aeruginosa* exhibited high chitinase activity levels, and in the present study, its nematicidal activity was enhanced by ~15% after the addition of 0.2% (m/v) colloid chitin in the growth medium (Fig. 1C), which indicated that chitinase would be a factor of nematicidal of *P. aeruginosa*. The chitinase pachi was cloned according to information from the *P. aeruginosa* genome (accession number: CP003149), and the sequencing results showed that pachi was similar to chitinase (99%, accession number: AFM65035)^23^. After being expressed in *E. coli*, pachi showed nematicidal activity and was selected for further experiments.

### Chitinases as high efficiency factors in bio-control

**Figure 2:**
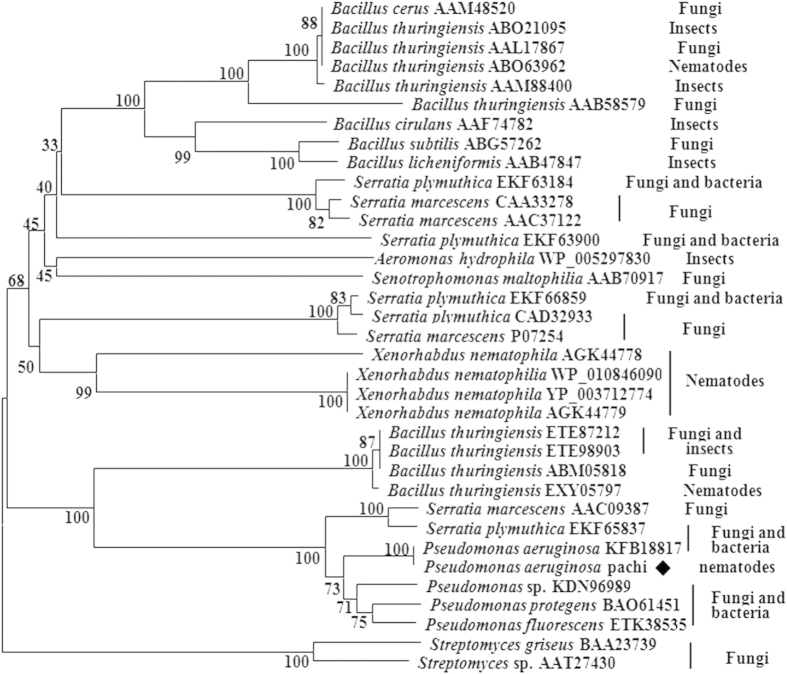
Phylogenetic analysis of the relationships among chitinases from different bacteria. The phylogenetic tree was constructed using the neighbor-joining method (MEGA6.0). All chitinase sequences were obtained from GeneBank and PDB (http://www.rcsb.org/pdb/home/home.do), and the accession numbers of chitinase were listed in the form of “AAM48520”, except for pachi. These chitinases were reported to infect multiple phytopathogenic organisms such as fungi, insects, and nematodes.

Figure 2 shows the phylogenetic analysis of the relationship among chitinases from different bacteria. Most chitinases were previously shown to have high antibiosis, antifungal, and insecticidal activities, but few have been reported as to be virulent to nematodes^7^,^24^.

### CHACry as a high nematicidal factor

**Figure 3:**
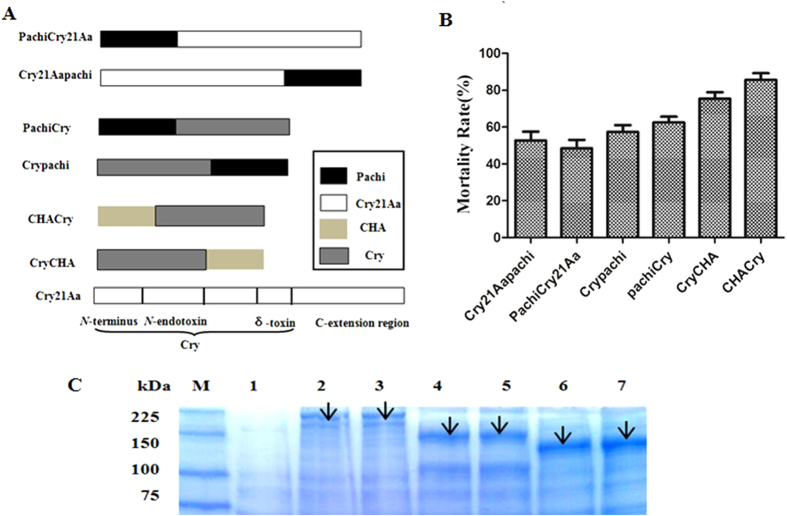
Schematic representation of construction, expression and nematicidal activity of fusion proteins. (**A**) Fusion protein construction. (**B**) Fusion protein tested on *C. elegans*. (**C**) Fusion protein expression. Lane M: Standard protein molecular mass marker (10–225.0 kDa). Lane 2–7: Induced cell lysate supernatants of pGEX-6p-1, pGEX-6p-Cry21Aapachi, pGEX-6p-pachiCry21Aa, pGEX-6p-Crypachi, pGEX-6p-pachiCry, pGEX-6p-CryCHA, and pGEX-6p-CHACry. The arrows point to target proteins.

In the present study, 6 fusion proteins were constructed and expressed in *E. coli* (Fig. 3A). The CHACry showed higher acitivity against *C. elegans* than others due to its high solubility (Fig. 3B), and thus it was selected for further experiments against *C. elegans*.

### Nematicidal activity analysis

**Figure 4:**
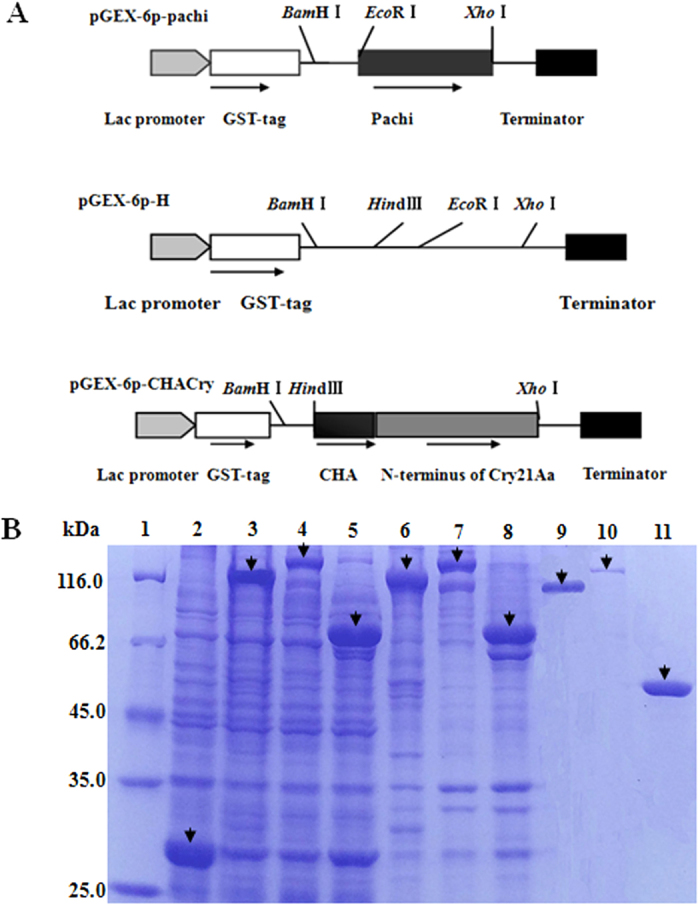
Recombinant plasmid construction and SDS-PAGE analysis of protein expression. (**A**) Fusion protein construction and partial restriction enzymatic site. (**B**) SDS-PAGE analysis of recombination protein expression in *E. coli* and purification. The arrows point to target proteins. Lane 1: The standard protein molecular mass marker (14.4–116.0 kDa). Lane 2–5: Induced cell lysate supernatants of pGEX-6p-1, pGEX-6p-CHACry, pGEX-6p-Cry21Aa, and pGEX-6p-pachi harbored in *E. coli*. Lane 6-8: Induced cell lysate sediments of pGEX-6p-CHACry, pGEX-6p-Cry21Aa, and pGEX-6p-pachi harbored in *E. coli*. Lane 9–11: Purified CHACry, Cry21Aa, and pachi proteins.

**Figure 5:**
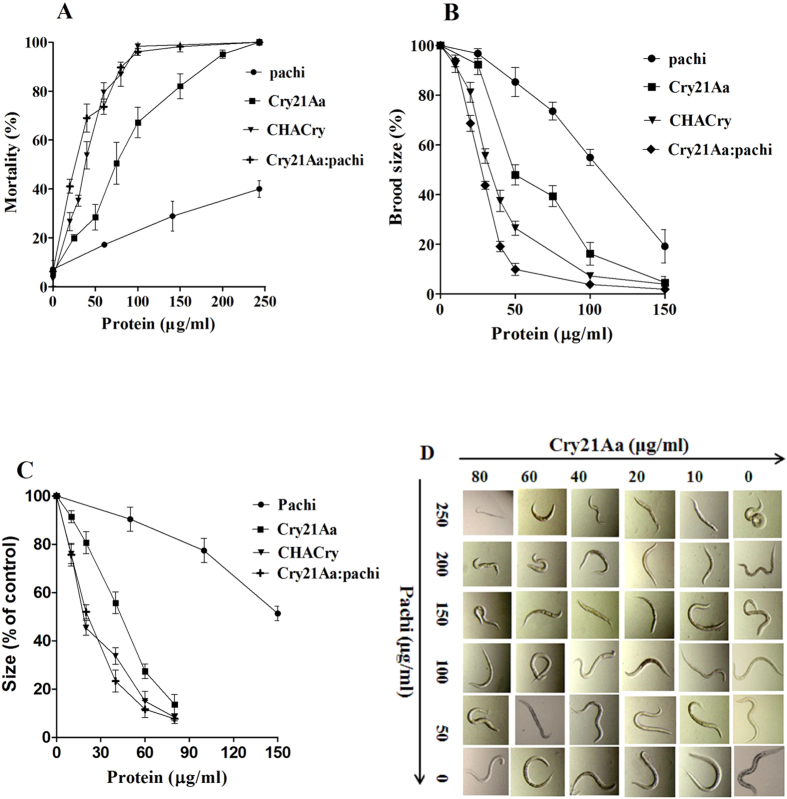
Pachi enhances Cry21Aa activity against *C. elegans*. (**A**) Mortality analysis was performed on L4 worms. (**B**) Brood size tests were performed on L4 worms. (**C**) Growth assays were performed on L1 larva. Error bars represent the standard deviation from the average values within three parallel experiments; (**D**) The effects of growth inhibition. All images were captured at 10X magnification under a Zeiss LSM 510 confocal microscope.

The effects of the proteins on mortality rate, brood size and growth of nematodes were systematically studied using purified protein (Fig. 4B), and pachi showed a nematicidal factor of LC_50_ = 387.3 ± 31.7 μg/ml (Fig. 5A and Table 1). Synergism was reported in previous studies in which chitinase showed enhanced activity against nematodes^11^,^19^,^25^. In the present study, pachi was mixed with Cry21Aa in different ratios (Table 1); pachi at a ratio of 3:1 with Cry21Aa significantly increased nematicidal activity. This combination resulted in an approximately 6.3-fold reduction in the LC_50_ compared with the expected value (SF, 6.3). Moreover, the mortality rate induced by the protein mixture at a ratio of 3:1 (LC_50_, 30.9 ± 4.1 μg/ml) was higher than that of pachi and Cry21Aa individually (LC_50_, 78.7 ± 5.1 μg/ml) (Fig. 5A). The brood size test and growth bioassay were systematically performed with each sample using pachi, Cry21Aa, Cry21Aa: pachi at a 1:3 ratio and the CHACry fusion protein. In the brood size analysis, pachi showed high activity against *C. elegans* breeding (IC_50_, 106.1 ± 4.3 μg/ml), and the cumulative effect of pachi:Cry21Aa (3:1) (IC_50_, 27.9 ± 0.9 μg/ml) showed a SF of 3.2 (Table 2 and Fig. 5B). In growth bioassay studies, the larvae grew much slower in the system containing pachi added to Cry21Aa. The protein mixture displayed higher activity against larval growth (GC_50_, 24.4 ± 3.3 μg/ml) than pachi (GC_50_, 154.7 ± 5.2 μg/ml) and Cry21Aa (GC_50_, 44.8 ± 2.3 μg/ml) individually (Fig. 5C,D). Additionally, the fusion protein CHACry (LC_50_, 37.8 ± 2.2 μg/ml; SF, 4.0) showed higher activity against *C. elegans* than the Cry21Aa:pachi mixture[1:1] (LC_50_, 44.0 ± 3.9 μg/ml; SF, 3.5). The same efficacy was observed for the brood size (IC_50_, 35.0 ± 1.7 μg/ml) test and the growth bioassay (GC_50_, 26.7 ± 4.9 μg/ml).

### Effects of pachi on egg shell, cuticle, and intestine

**Figure 6:**
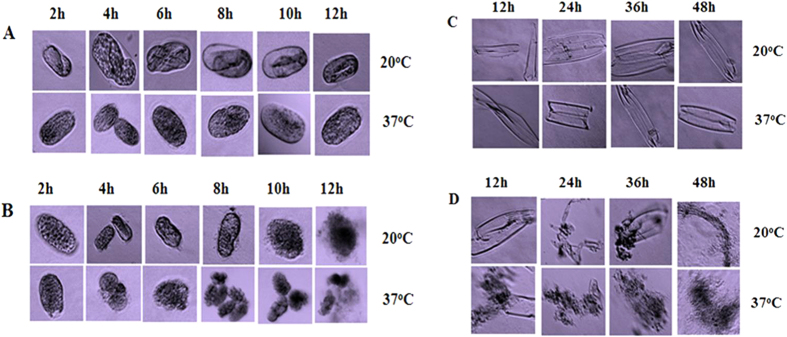
Egg shell and cuticle digestion by pachi. (**A**) Eggs were incubated with PBS; (**B**) Eggs were incubated with pachi; (**C**) Cuticles were incubated with PBS; (**D**) Cuticles were incubated with pachi. Images were taken under a Zeiss LSM 510 confocal microscope at 20X magnification.

In the present study, we observed that pachi acts effectively on the egg shell and cuticle to kill nematodes. Fresh eggs were incubated with pachi at 20 °C and 37 °C for different time periods (2, 4, 6, 8, 10, 12 h). The egg surface began to show roughness and irregularities after 6 h (at 20 °C), whereas eggs incubated with the control (PBS) were smooth and remained formed after 6 h (Fig. 6A). The egg shells were destroyed in 4 h when incubated with pachi at 37 °C and disappeared after 12 h of incubation, whereas eggs incubated in the PBS control remained unaffected (Fig. 6B).

**Figure 7:**
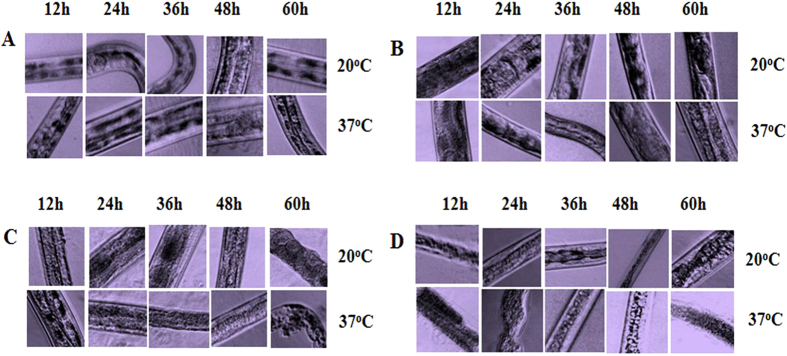
Effects of Cry21Aa, pachi, and CHACry on the intestine. (**A**) Worms were incubated with PBS; (**B**) Worms were incubated with Cry21Aa; (**C**) Worms were incubated with pachi; (**D**) Worms were incubated with CHACry. Images were taken under a Zeiss LSM 510 confocal microscope at 20X magnification.

Similarly, the nematode cuticle was critically destroyed after incubation with pachi at 37 °C for 24 h, with hardly any pieces of whole cuticle observed under a microscope after 48 h (Fig. 6D). In contrast, the cuticle remained in its original state after 48 h of incubation in PBS (Fig. 6C). Furthermore, most ICPs were able to destroy the nematode intestine^8^. After incubation with pachi for 24 h, the intestine was digested and destroyed, leading to nematode death (Fig. 7C). The intestine shrunk and became difficult to identify after incubation with Cry21Aa for 36 h, whereas the cuticle remained unaffected (Fig. 7B). Moreover, the ability of Cry21Aa to destroy the intestine of nematodes was enhanced by pachi (Fig. 7D). When the catalytic domain of pachi was fused with the Cry21Aa toxin region, the intestine and the cuticles were destroyed after 60 h of incubation at 37 °C. This result was probably due to each protein killing the nematodes by different mechanisms. Here, the degradation of the cuticle and intestine demonstrated that pachi damaged the skin and intestine, whereas the ICP acted only on the intestine of the nematode.

## Discussion

In various previous studies, *P. aeruginosa* was reported to cause disease in insects, nematodes, and mice^18^,^26^,^27^,^28^. In the present study, we attempted to explore new nematicidal agents from *P. aeruginosa*. Chitinase from *P. aeruginosa* has been selected not only because of the regulation of its nematicidal activity by colloidal chitin (Fig. 1C) but also the scarcity of reports on chitinase as a bio-control agent for nematodes (Fig. 2). The chitinase pachi showed a high potential to kill nematodes, bio-control broods, and inhibit growth through digestion of the cuticle, egg shells, and intestine of *C. elegans.* Moreover, the chitinase pachi enhanced Cry21Aa activity against *C. elegans*.

Chitin played an important role in the formation of the egg shell and cuticle of *C. elegans* by acting as an important barrier to protect nematodes from infection by pathogenic microorganisms^14^. Chitinase, due to its high chitin-degrading activity, has been used as a bio-control agent against phyto-pathogenic bacteria, fungi, insects, and nematodes (Fig. 2). Several bacteria such as *Bacillus*, *Serratia*, *Pseudomonas*, and *Streptomyces* could use chitin as a carbon source and infect hosts using chitinase^29^,^30^,^31^,^32^. The antifungal mechanism of chitinase was due to the hydrolysis of chitin in the cell wall, leading to cell lysis^24^. Chitinase could also destroy the intestine and cuticle of insects by digesting chitin, leading to gut shrinkage and osmotic pressure imbalance^33^. In this study, pachi effectively destroyed the *C. elegans* cuticle and intestine, resulting in the death of nematodes (Figs 6C,D and 7). Pachi also displayed high activity for the digestion of egg shells and the inhibition of egg hatching, which was supported by a study carried out by Gan *et al.*^34^. In the study of Mercer *et al.*^35^, the chitinase increased hatch rates of nematode eggs but caused the death of juveniles instantly. In the present study, chitin upregulated the activity of chitinase of *P. aeruginosa* and increased its nematicidal activity. Chitin could act as important carbon source and signal molecules to upregulate production of chitinase. However, thus far, the information about the nematicidal mechanism of chitinase is very limited in the literature^7^. The complex structure and multi-component system of chitin has made elusive the understanding of whether the mechanism of action of chitinase is different or the same in fungi, insects, and nematodes.

In this study, two different properties proteins, pachi obtained from *P. aeruginosa* and Cry21Aa from *B. thuringiensis*, functioned together as a nematicidal agent. Previous studies showed that chitinase could enhance the insecticidal activity of *B. thuringiensis* by penetrating the peritrophic membrane barrier in the larval midgut and aiding the ICPs to bind their receptors on epithelial cell membranes^36^. To enhance its large-scale application and use as an insecticidal agent, *B. thuringiensis* was used as a host for co-expressing several chitinases from *Bacillus licheniformis*, *Bacillus circulans*, *Pseudomonas maltophilia*, *Bacillus sphaericus*, *Aeromonoas hydrophila*, *Serratia marcescens*, and *Nicotiana tabacum*^22^,^37^,^38^,^39^,^40^,^41^. Moreover, nematodes had a similar peritrophic membrane structure and composition as insects^42^. Therefore, as reported, the chitinase pachi also could enhance the toxicity of Cry21Aa against *C. elegans* by the similar modes of insecticidal.

Another attractive property was that the CHACry fusion protein showed higher activity than each individual protein. Six fusion proteins were constructed and tested on *C. elegans*, but the CHACry showed highest activity and solubility. A previous study indicated that C-terminal extension region was related to solubility of protein, and the CHA had higher solubility than pachi^23^. Recently, many fusion proteins have been constructed and co-expressed to enhance relative activity, and more data about such fusion proteins would facilitate the access to the binding sites, improve their stability and broaden the insect-resistance spectrum^43^,^44^. Fusion venoms were well known to resist proteolytic activity in insects, alter the shape of the original protein crystals and alter receptor binding sites present in the midgut of insects^45^. Previous studies also showed that the ICPs from *Bacillus* expressed in *E. coli* easily formed inclusion bodies and lowered the activity due to the dissolution process that was necessary for ICP activation^46^. Our previous study indicated that solubility of chitinase was increased after removing the chitin binding domain^23^. In the present study, the catalytic domain of pachi was fused with the toxin region of Cry21Aa, and the CHACry fusion protein (~50% soluble) showed better solubility than Cry21Aa, which increased Cry21Aa activity against *C. elegans* (Fig. 4B). Additionally, each toxin had a unique mechanism of killing hosts and damaging the host cell^11^. Many researchers believed that the ICP damaged the intestine of the host and that chitinases hydrolyzed the cuticle and egg shell^35^,^47^. Our results revealed that the chitinase showed high intestinal digestion activity, which would protect the ICP from proteolysis and help the ICP bind to its receptor. Moreover, the chitinase also showed high cuticle degradation activity and provided a new way for the ICP to enter into the intestine of the nematode. However, the mechanism of synergy by which a biopesticide entered the nematode and became resistant to insecticidal attack remained to be elucidated.

Previous studies indicated that *P. aeruginosa* PA14 work in two different modes to kill *C. elegans*. At low salt concentration medium, PA14 showed a mild infection and killed the nematodes in 72–96 h (‘slow killing’) via production of hydrolytic enzyme, like protease, which was due to the mass accumulation of bacteria in the intestine and enzymatic degradation. But at high salt medium, PA14 produced diffusible toxins like cyanongen, phenazines, and pycoyanin, and killed nematodes within a short time period (‘fast killing’) by inhibiting metabolic pathways^48^. In this study, *P. aeruginosa* killed *C. elegans* also by producing hydrolytic enzyme (chitinase) and toxin. It was interesting to note that the strain (*P. aeruginosa*) did not produce protease, which was different from some previous studies^17^, while chitinase played a vital role in nematicidal activity. Recent studies of *Pseudomonas* infection in nematodes were based upon the quorum sensing system (QS)^49^. It was a complex cell-to-cell signaling system allowing the bacteria to sense and regulated their own cell densities. There were three types of secretion systems (type I, type II and type III) in QS of *P. aeruginosa* to secrete extracellular hydrolytic enzyme into the cytoplasm of hosts^50^. Type II secretion system regulated the production of extracellular hydrolases, like elastase, alkaline phosphatase, phospholipase C, and chitinase etc.^51^. In the present study, the genome analysis of *P. aeruginosa* predicted that some extracellular hydrolases (like serine protease, collagenase, phospholipase C, chitinase) might play an important role in killing nematodes. The mechanisms of different hydrolases in killing nematodes would be an interesting topic in future studies.

## Materials and Methods

### Strains and culture

The strains and plasmids used in this study are listed in Supplementary data Table 1. The bacterial strain was isolated from a mud soil sample from South Lake near Huazhong Agricultural University, Wuhan, China. The culture was grown in nutrient agar medium containing peptone 1%, yeast extract 0.5%, NaCl 1%, and agar 1.5% (m/v). The selected bacterial strain was identified as *P. aeruginosa* by 16S rRNA sequencing. *Escherichia (E.) coli* strains were maintained in Luria-Bertani medium containing ampicillin (100 μg/ml). All bacterial strains were kept in 20% (v/v) glycerol suspension at −80 °C. The *C. elegans* N2 wild-type strain was provided by the Caenorhabditis Genetics Center (CGC) and maintained on nematode growth medium (NGM) agar plates with *E. coli* OP_50_ as its food at 20 °C and stored at −80 °C.

### Chitinase activity

*P. aeruginosa* was collected and resuspended in PBS buffer (final OD_600_ ~ 3.0), followed by ultrasonic disruption. The supernatant was selected to test its chitinase and nematicidal activity. Chitinase activity was measured according to method described by Chen *et al.*^23^, while nematicidal activity was measured according to the method of Bischof *et al.*^52^.

### Cloning of gene pachi and Cry21Aa

Genomic DNA extracted from *P. aeruginosa* was used as a template to amplify pachi using the primers pachi-f and pachi-r (Supplementary data Table 2). The Cry21Aa gene was amplified from the plasmid pHT304-Cry21Aa using the primer pair Cry21Aa-f and Cry21Aa-r. The *pachi* gene was digested by *Eco*R I and *Xho* I and cloned into the pGEX-6p-1 vector to construct the pGEX-6p-pachi expression vector, whereas *Cry21Aa* was digested by *Bam*H I and *Xho* I to construct the pGEX-6p-Cry21Aa expression vector. The recombinant plasmids were then transformed into *E. coli* BL21 (DE3) cells.

### Construction of fusion proteins

The pGEx-6p-1 plasmid was used as the template to construct the pGEX-6p-H plasmid by inserting an additional *Hin*d III restriction enzyme site between *Bam*H I and *Eco*R I using the primer pair 6p-H-f and 6p-H-r. Rapid polymerase chain reaction (PCR)-based site-directed mutagenesis was used to add the two restriction enzyme sites. Fusion proteins were constructed by using over-lapping PCR. The CHACry (3087 bp; 114.5 kDa; 3 bp for termination codon) was constructed by fusing the catalytic domain of pachi (1026 bp; 37.7 kDa) with the toxin region of Cry21Aa (Cry) (2058 bp; 76.8 kDa). The Cry contained the N-terminus extension region, endotoxin N and delta-endotoxin C. Cloning of the Cry21Aa N-terminus was directed by using the primer pair Cry21Aa-f_2_ and Cry21Aa-r_2_. The catalytic domain (CHA) of pachi was cloned with the primer pair CHA-f and CHA-r. Finally, the recombinant *CHACry* gene was cloned with the primer pair CHA-f and Cry21Aa-r_2_ by overlapping PCR. Recombinant pGEX-6p-CHACry was transformed into *E. coli* BL21 (DE3) cells to express the CHACry fusion protein.

### Protein expression and purification

*E. coli* BL21 (DE3) cells harboring pGEX-6p-pachi, named DE3/pGEX-6p-pachi, were inoculated into LB broth (ampicillin; 100 μg/ml) with shaking at 37 °C for 12 h. The seed culture was used to inoculate production broth (v/v, 2/100), and growth was induced by adding IPTG (1 mM) after 2–3 h. The IPTG-induced production broth was incubated at 18 °C for 14 h with shaking (250 rpm/min).

Finally, the induced cells were collected by centrifugation, resuspended in phosphate-buffered saline (PBS) buffer (NaCl 0.8%, KCl 0.02%, Na_2_HPO_4_ 0.14%, KH_2_PO_4_ 0.03%; pH 7.0) and homogenized using a high-pressure homogenizer (NS100IL 2K, Niro Soavi, Germany). The target proteins (Cry21Aa, pachi and CHACry) were purified using a glutathione S-transferase (GST) Gene Fusion System (GE Healthcare, USA) and eluted from the GST tag by 3C proteases (PreScission, Pharmacia). The molecular weight was analyzed by SDS-PAGE with 10% polyacrylamide gels. The protein concentration was measured by the Bradford method using bovine serum albumin (BSA) as a standard.

### Quantitative analysis of nematicidal activity

The purified proteins were used for bioassays including quantitative mortality tests, brood size assays, and growth analyses. The bioassay procedures and 50% lethal/inhibition/growth concentration (LC_50_/IC_50_/GC_50_) evaluations were undertaken according to the method of Bischof *et al.*^52^.

### Cuticle and egg shell digestion

The cuticle was separated from the nematode body as described by Cox *et al.*^14^. Cuticles (eggs) was incubated with pachi at 20 °C and 37 °C for various time periods. The L4 worms were incubated with pachi, Cry21Aa, and the CHACry fusion protein at 20 °C and 37 °C for various time periods. Pachi was used to study complete digestion at 1 mg/ml for the cuticle and at 200 μg/ml for the egg shell and intestine. The effects of Cry21Aa on the intestine were assessed at a final concentration of 150 μg/ml, while CHACry was assessed at 100 μg/ml. Pachi (1 mg/ml) was used as an effective nematicidal dose to ensure the complete killing of *C. elegans*.

### Synergistic activity assays for pachi and Cry21Aa in L4 worms of the N2 strain

The synergistic factor was calculated by the formula of Tabashnik *et al.*^53^: 1/LC_50(m)_ = Ra/LC_50(a)_ + Rb/LC_50(b)_, where Ra and R_b_ indicate the percentage of toxin A and toxin B proteins used in the final mixture; LC_50(a)_ and LC_50(b)_ represent the LC_50_ values for each toxin, and LC_50(m)_ is the expected theoretical value of LC_50_ calculated from the formula above. The real LC_50_ value was calculated from the bioassay for the observed toxicity of the mixture. Synergism was indicated with a SF value of greater than 1.

### Statistical analysis

Independent experiments were repeated at least three times. All of the data obtained were analyzed using GraphPad Prism 5.0 and Excel 2003 software for figures and LC_50_ values. All of the values were expressed as the mean values ± standard deviation, with the statistical significance set at p < 0.05.

### Image analysis by confocal microscopy

To study the effect of each protein on nematodes and their body parts, images were captured using a 20X objective lens with confocal laser scanning microscopy on a Zeiss LSM 510 microscope (CLSM; Zeiss LSM 510) imaging. Mixture of diethyl ether/ethanol absolute (1:1) was used as anaesthetic treatment to keep worms static during image capture. All pictures were processed using Photoshop 7.0 software, and the worm sizes were calculated using ImageJ 2.4.1.7.

### Nucleotide sequence accession number

The nucleotide sequences of *Pseudomonas aeruginosa* 16S and pachi have been submitted to GeneBank under accession numbers KR007310 (16S) and KR007311 (pachi).

## Additional Information

**How to cite this article**: Chen, L. *et al.* Enhanced nematicidal potential of the chitinase *pachi* from *Pseudomonas aeruginosa* in association with *Cry21Aa*. *Sci. Rep.* **5**, 14395; doi: 10.1038/srep14395 (2015).

## Supplementary Material

Supplementary Information

## Figures and Tables

**Table 1:** Synergism and elevation in activity of pachi and Cry21Aa.

**Nematicidal activity**				
**Toxin**	**Regression coefficient**	**LC_50_ (μg/ml)**^a^		**Synergistic factor (SF)**
		**Actual value**	**Expected value**	
pachi	0.9651	387.3 (355.6–419.1)		
Cry21Aa	0.9708	78.7 (73.2–84.2)		
Combination protein(ratio)				
Cry21Aa:pachi(2:1)	0.9660	45.7 (41.9–49.5)	108.7	2.4
Cry21Aa:pachi(1:1)	0.9663	44.0 (40.2–47.9)	154.3	3.5
Cry21Aa:pachi(1:2)	0.9591	43.5 (39.2–47.8)	168.0	3.9
Cry21Aa:pachi(1:3)	0.9544	30.9 (26.8–35.1)	195.1	6.3
Cry21Aa:pachi(1:5)	0.9333	39.9 (34.5–45.4)	234.4	5.9
Cry21Aa:pachi(1:8)	0.9617	53.3 (49.0–57.7)	269.5	5.1
Fusion protein CHACry	0.9805	37.8 (35.6–40.0)	154.3	4.1

^a^The real values were calculated as described by Bischof *et al.*^52^ with statistical significance set at p < 0.05. Expected values were calculated using the equation of Tabashnik *et al.*^53^.

**Table 2:** Chitinase enhanced Cry21Aa activity against *C. elegans.*

**Toxin**	**Bio-control**		
	**LC_50_ (μg/mL)**	**IC_50_ (μg/mL)**	**GC_50_ (μg/mL)**
pachi	387.3 (355.6–419.1)	106.1 (101.8–110.3)	154.7 (149.5–159.9)
Cry21Aa	78.7 (73.2–84.2)	60.2 (54.1–66.3)	44.8 (42.4–47.1)
Cry21Aa:pachi(1:3)	30.9 (26.8–35.1)	27.9 (27.0–29.0)	24.4 (21.1–27.7)
Fusion protein CHACry	38.8 (36.6–41.0)	35.0 (33.4–36.7)	26.7 (21.8–31.6)
