# Supplementary material for: Enhanced nematicidal potential of the chitinase pachi from Pseudomonas aeruginosa in association with Cry21Aa
Source: Sci Rep. 2015 Sep 24;5:14395. doi: 10.1038/srep14395 (PMC4585872; doi:10.1038/srep14395)
Supplement: Supplementary Information [file srep14395-s1.docx]

**Enhanced nematicidal potential of the chitinase *pachi* from *Pseudomonas aeruginosa* in association with *Cry21Aa***

***Lin Chen^1^, Huang Jiang^1^, Qipeng Cheng^1^, Junpeng Chen^1^, Gaobing Wu^2^, Ashok Kumar^1^, Ming Sun^1^, Ziduo Liu^1,^****

*^1^ State Key Laboratory of Agricultural Microbiology, College of Life Science and Technology, Huazhong Agricultural University, Wuhan 430 070, China*

*^2^ State Key Laboratory of Agricultural Microbiology, College of Plant Science and Technology, Huazhong Agricultural University, Wuhan 430070, China*

*Corresponding author:* [**Ziduo Liu];** E-mail: [lzd@mail.hzau.edu.cn](mailto:lzd@mail.hzau.edu.cn)

Tel: 86-15927372165; Fax: 86-27-87280670

**Table 1. Model organism, bacterial strains and plasmids**

| Strain/plasmid(s) | Characteristics | Origin |
| --- | --- | --- |
| Strains |  |  |
| *C. elegans* | Bristol (N2), the wild-type strain | -^b^ |
| *E. coli* |  |  |
| DH5α | F^-^,φ80d lacZΔM15; for harboring plasmid | -^a^ |
| BL21 (DE3) | T7 promoter; for protein expression | -^a^ |
| *P. aeruginosa*. | Wild type; containing gene pachi | This study |
| Plasmids |  |  |
| pGEX-6p-1 | ori *E. coli* (Amp^r^); containing GST-tag for purification; 4.9 kb | -^a^ |
| pHT304-Cry21Aa | From pHT304; containing Cry21Aa; Erm^r^ | -^b^ |
| pGEX-6p-pachi | From pGEX-6p-1; containing pachi; Amp^r^ | This study |
| pGEX-6p-Cry21Aa | From pGEX-6p-1; containing Cry21Aa; Amp^r^ | This study |
| pGEX-6p-H | From pGEX-6p-1; containing new restriction sites *Hind* Ⅲ; Amp^r^ | This study |
| pGEX-6p-CHACry | From pGEX-6p-H; containing fusion protein CHACry; Amp^r^ | This study |

^a^ Stored at State Key Laboratory of Agricultural Microbiology, College of Life Science and Technology, Huazhong Agricultural University, Wuhan, Hubei, China.

^b^ Provided by Prof. Ming Sun (m98sun@mail.hzau.edu.cn.) and stored at State Key Laboratory of Agricultural Microbiology, College of Life Science and Technology, Huazhong Agricultural University, Wuhan, Hubei, China.

**Table 2. List of primers**

| Primer | 5’ to 3’ | Purpose |
| --- | --- | --- |
| pachi-f | CCGGAATTCATGATCAGGATCGACTTTTCCCAGTTGCA | Cloning pachi |
| pachi-r | CCGCTCGAGTCAGCGCAGCGGCCGCC | Cloning pachi |
| Cry21Aa-f | CGCGGATTCATGACAAATCCAACTATAC | Cloning Cry21Aa |
| Cry21Aa-r | CCGCTCGAGTTACTCTTCTATGATTTC | Cloning Cry21Aa |
| 6p-H-f | CCGAAGCTTCCGGAATTCCCGGGTCGACTC | Adding restriction enzyme sites |
| 6p-H-r | CGGAAGCTTCGGGGATCCCAGGGGCCCCTG | Adding restriction enzyme sites |
| CHA-f | CCCAAGCTTATGATCAGGATCGACTTTTCCCAGTTGC | Cloning catalytic domain of pachi |
| CHA-r | AGTTGGATTTGTCATGCGCTGGTCGCCGCC | Cloning catalytic domain of pachi |
| Cry21Aa-f_2_ | GGCGGCGACCAGCGCATGACAAATCCAACT | Cloning N-terminus of Cry21Aa |
| Cry21Aa-r_2_ | CCGCTCGAGTCAAATAAATTCAATACGATCTAAAAAAAGA | Cloning N-terminus of Cry21Aa |

Underlined bases represent restriction enzyme sites; f, forward; r, reverse
